# Two new wood-decaying fungi, *Resupinatus tropicus* and *Scopuloides hainanensis* (Agaricomycetes, Basidiomycota) from Hainan Province, southern China

**DOI:** 10.3897/mycokeys.133.198088

**Published:** 2026-06-01

**Authors:** Qian-Xin Guan, Heng Zhao, Fang Wu, Xin Zhang, Yu-Cheng Dai, An-Hong Zhu

**Affiliations:** 1 School of Ecology and Nature Conservation, Beijing Forestry University, Beijing 100083, China Coconut Research Institute, Chinese Academy of Tropical Agricultural Sciences Hainan China; 2 CAS Key Laboratory of Forest Ecology and Silviculture, Institute of Applied Ecology, Chinese Academy of Sciences, Shenyang 110164, China CAS Key Laboratory of Forest Ecology and Silviculture, Institute of Applied Ecology, Chinese Academy of Sciences Shenyang China; 3 Coconut Research Institute, Chinese Academy of Tropical Agricultural Sciences, Wenchang 571339, Hainan, China School of Ecology and Nature Conservation, Beijing Forestry University Beijing China

**Keywords:** Agaricales, new taxon, Polyporales, taxonomy, wood-inhabiting fungi

## Abstract

Wood-decaying fungi are among the most important groups of macrofungi with crucial ecological roles and economic values. In this study, two new wood-decaying fungal species, *Resupinatus tropicus* and *Scopuloides hainanensis*, are described from Hainan, southern China, based on the morphological and phylogenetic approaches. *Resupinatus tropicus* is characterized by sessile, gelatinous, flabelliform or occasional suborbicular basidiomata with lamellate hymenophore, a monomitic hyphal system bearing clamp connections on generative hyphae, the presence of cheilocystidia and pleurocystidia, and cylindrical to oblong-ellipsoid basidiospores. *Scopuloides hainanensis* is characterized by resupinate, membranaceous basidiomata with grandinioid, white to gray hymenial surface, a monomitic hyphal system with simple-septate generative hyphae, the presence of subulate to fusiform lamprocystidia, and subcylindrical to allantoid basidiospores. The phylogenetic analyses showed that *Resupinatus abieticola*, *R. alboniger*, *R. americanus*, and *R. tropicus* grouped together to form a distinct and well-supported clade using ITS + nLSU genetic loci. *Scopuloides hainanensis* was closely related to *S. hydnoides* and *S. yunnanensis* with strong support. A full description, illustrations, and phylogenetic analyses results of the two new species are provided. In addition, keys to the known species of *Resupinatus* and *Scopuloides* in China are presented.

## Introduction

Hainan Province, located in southern China, contains the most extensive and well-preserved tropical forests. Hainan Tropical Rainforest National Park provides a wide niche for fungi (Dai et al. 2011; Li et al. 2024; Qin et al. 2025; Zeng et al. 2026). Among these diverse fungal groups, wood-decaying fungi represent one of the most important groups of macrofungi, playing essential roles in energy flow and regeneration within forest ecosystems (Dai 2010; Zhao et al. 2015; Dai et al. 2021; Wang et al. 2023; Dong et al. 2024; Zhao et al. 2025a, 2026). Recently, the diversity of Chinese macrofungi has been extensively studied and a large number of new taxa for China have been discovered, among which wood-decaying fungi are an important component (Liu et al. 2022, 2025; Wu et al. 2022a; Zhang et al. 2023; Zhou et al. 2023; Dong et al. 2024, 2025; Zheng et al. 2024; Zhou et al. 2024; Wijesinghe et al. 2025; Yang et al. 2025; Zhao et al. 2025a, 2025b; Deng et al. 2026; Wang et al. 2026; Yuan et al. 2026; Zhuang et al. 2026a, 2026b). For example, Dai et al. (2021) reported that Hainan ranks second in China for published new fungal species, with 110 species after Yunnan Province, subsequently increased to 264 (Zhao et al. 2025a). Additionally, Ma et al. (2022) documented that wood-decaying macrofungi in Hainan comprise 19 orders, 68 families, 256 genera, and 702 species, which mainly belong to Agaricales, Hymenochaetales, Polyporales, and Xylariales. These studies indicate that Hainan not only harbors a rich diversity of known wood-decaying fungi but also suggest a high potential for unknown species.

The genus *Resupinatus* Nees ex Gray was proposed with the type species *R. applicatus* (Batsch) Gray, which belongs to Resupinataceae, Agaricales, Agaricomycetes, within Basidiomycota (Hyde 2024; Vizzini et al. 2024). The genus is characterized by small, pleurotoid to cupulate lamellate basidiomata, while cyphelloid, poroid, and merulioid forms are also included in the genus (Gray 1821; Singer 1986; Redhead and Nagasawa 1987; Bodensteiner et al. 2004; Thorn et al. 2005; McDonald 2015; Bijeesh et al. 2020; Carpouron et al. 2024; Triantafyllou et al. 2026; Zhuang et al. 2026b). In recent years, some new species, such as *R. abieticola* Triantafyllou & Gonou-Zagou, *R. angulatus* Q.X. Guan et al., *R. latemarginatus* Q.X. Guan et al., *R. porrigens* J.Z. Xu & Yu Li, *R. reviviscens* Carpouron & Raspé, *R. sinoapplicatus* Q.X. Guan et al., *R. sinuosus* Q.X. Guan et al., *R. tenuis* J.H. Dong & C.L. Zhao, and *R. yunnanensis* Yang Yang & C.L. Zhao, were proposed based on a combination of morphological features and molecular evidence (Yang et al. 2023; Carpouron et al. 2024; Liu et al. 2024; Dong et al. 2025; Triantafyllou et al. 2026; Zhuang et al. 2026b). Currently, a total of 58 species of *Resupinatus* is accepted, based on the MycoBank database (http://www.MycoBank.org, accessed on 30 April 2026) and the Index Fungorum (http://www.indexfungorum.org, accessed on 30 April 2026). Among them, 10 known species are recorded in China, viz. *R. alboniger* (Pat.) Singer, *R. angulatus*, *R. applicatus*, *R. latemarginatus*, *R. porrigens*, *R. sinoapplicatus*, *R. sinuosus*, *R. tenuis*, *R. trichotis* (Pers.) Singer, and *R. yunnanensis* (Li and Bau 2014; Yang et al. 2023; Liu et al. 2024; Dong et al. 2025; Zhuang et al. 2026b).

The genus *Scopuloides* (Massee) Höhn. & Litsch., typified by *S. hydnoides* (Cooke & Massee) Hjortstam & Ryvarden, is a monophyletic genus of Meruliaceae (Polyporales, Chen et al. 2021). Macroscopically, *Scopuloides* species typically have ceraceous or membranaceous basidiomata, with white to buff and odontioid, hydnoid, or grandinioid hymenophores. Microscopically, the genus is characterized by a compact subiculum with agglutinated, short-celled subicular hyphae, short basidia, small basidiospores, and the presence of lamprocystidia (Wu 1990; Gilbertson and Nakasone 2003; Bernicchia and Gorjón 2010; Gu et al. 2025). Currently, a total of 11 species of *Scopuloides* is accepted, based on the MycoBank database (accessed on 20 April 2026) and the Index Fungorum (accessed on 20 April 2026). Among them, eight known species are distributed in China, viz. *S. allantoidea* C.C. Chen & Sheng H. Wu, *S. dimorpha* (Sang H. Lin & Z.C. Chen) C.C. Chen & Sheng H. Wu, *S. ellipsoidea* S.H. He et al., *S. farinacea* Z.R. Gu & C.L. Zhao, *S. grandinioides* S.H. He et al., *S. hydnoides* (Cooke & Massee) Hjortstam & Ryvarden, *S. rimosa* (Cooke) Jülich, and *S. yunnanensis* Z.R. Gu & C.L. Zhao (Chen et al. 2021; Gu et al. 2024, 2025).

During investigations of wood-decaying fungi in Hainan Province, China, four specimens were collected, representing two undescribed species based on morphological and phylogenetic analyses, and they are identified as new species in *Resupinatus* and *Scopuloides*, respectively.

## Materials and methods

### Morphological studies

During the sampling trips to the Hainan, southern China, four wood-decaying fungi specimens were collected from dead wood. The voucher specimens were deposited in the herbarium of the Institute of Microbiology, Beijing Forestry University (BJFC). The morphological descriptions are based on field notes and voucher specimens, following the methods outlined in previous studies (Mao et al. 2023; Liu et al. 2025). For micro-morphological data, a light microscope was used to examine dried specimens. Sections were observed at magnifications up to 1000× using a Nikon Eclipse 80i microscope with phase contrast illumination (Nikon, Tokyo, Japan). Measurements, descriptions, and illustrations of microscopic features were derived from preparations mounted in Cotton Blue, KOH (5%), Phloxine B (2% C_20_H_4_Br_4_Cl_2_K_2_O_5_), and Melzer’s reagent. To account for natural variability, the upper and lower 5% of extreme values were excluded from each dataset, with the adjusted ranges presented in parentheses. The following abbreviations are used: KOH = 5% potassium hydroxide; IKI = Melzer’s reagent; IKI– = neither amyloid nor dextrinoid; CB = Cotton Blue; CB– = acyanophilous; CB+ = cyanophilous; L = mean spore length; W = mean spore width; Q = length-to-width ratio; (n = x/y), where x is the number of spores measured and y is the number of specimens examined. Color descriptions follow Anonymous (1969) and Petersen (1996).

### DNA extraction, PCR, and sequencing

DNAs were extracted from dried voucher specimens using a CTAB rapid kit (DN14, Aidlab Biotechnologies Co., Ltd., Beijing). The 25 μL polymerase chain reaction (PCR), including12.5 μL of PCR mix, 9.5 μL of ddH_2_O, 1 μL of forward primers, 1 μL of reverse primers, and 1 μL of DNA template, were performed according to the manufacturer’s instructions. The internal transcribed spacer (ITS) and large subunit nuclear ribosomal RNA gene (nLSU) were amplified using the ITS5/ITS4 (White et al. 1990) and LROR/LR7 (https://sites.duke.edu/vilgalyslabrdna_primers_for_fungi/), respectively.

The PCR procedure for ITS and nLSU was as follows: initial denaturation at 95 °C for 3 min, followed by 34 cycles at 94 °C for 40 s, annealing at 54 °C for 45 s, and extension at 72 °C for 1 min, with a final extension at 72 °C for 10 min. The PCR products were purified and sequenced at the Beijing Genomics Institute (BGI), China, with the same primers as used in PCR. Newly generated sequences were deposited in GenBank. All sequences analyzed in this study are listed in Suppl. material 1.

### Phylogenetic analyses

In the present study, the phylogenetic relationships of *Resupinatus* were reconstructed using the concatenated dataset (ITS + nLSU), and two specimens of *Hohenbuehelia* Schulzer were used as the outgroups (Zhuang et al. 2026b). The phylogeny of *Scopuloides* was constructed based on dataset composed of concatenated ITS + nLSU sequences with the members of *Ceriporiopsoides* C.L. Zhao as outgroups (Gu et al. 2025). Sequence alignments for each locus were performed independently using MAFFT v7 (Katoh and Standley 2013), after which ambiguously aligned regions were excluded. The aligned ITS and nLSU datasets were concatenated in PhyloSuite v1.2.3 (Zhang et al. 2020) prior to phylogenetic reconstruction. The most optimal nucleotide substitution model for the combined dataset was determined using ModelTest-NG v0.1.7 (Darriba et al. 2020).

Phylogenetic relationships were inferred using both Maximum Likelihood (ML) and Bayesian Inference (BI) methods, implemented in RAxML v8 (Stamatakis 2014) and MrBayes v3.2.6 (Ronquist et al. 2012), respectively, following previously established procedures (Nie et al. 2025; Song et al. 2025). Maximum Likelihood analyses were conducted with 1,000 bootstrap replicates under the selected best-fit model. The Bayesian Inference were conducted for 2,000,000 generations for *Resupinatus* and *Scopuloides*, with two independent runs, each consisting of four Markov chains (one cold and three heated). Trees were sampled every 1,000 generations, resulting in a total of 2,000 sampled trees. The first 25% of trees (500 trees) were discarded as burn-in, and the posterior distribution was therefore estimated based on the remaining 1,500 trees. The burn-in fraction of 25% was determined based on the inspection of likelihood convergence and stationarity of parameter traces of MrBayes v3.2.6. Convergence between runs was further assessed by ensuring that the average standard deviation of split frequencies was below 0.01. In addition, base frequencies were estimated during the analysis under the selected substitution model. The phylogenetic trees were visualized using FigTree v1.4.4. Nodes with ML bootstrap support values and BI posterior probabilities greater than or equal to 70% and 0.90 were considered as significantly supported.

## Results

### Phylogeny

**Figure 1.**
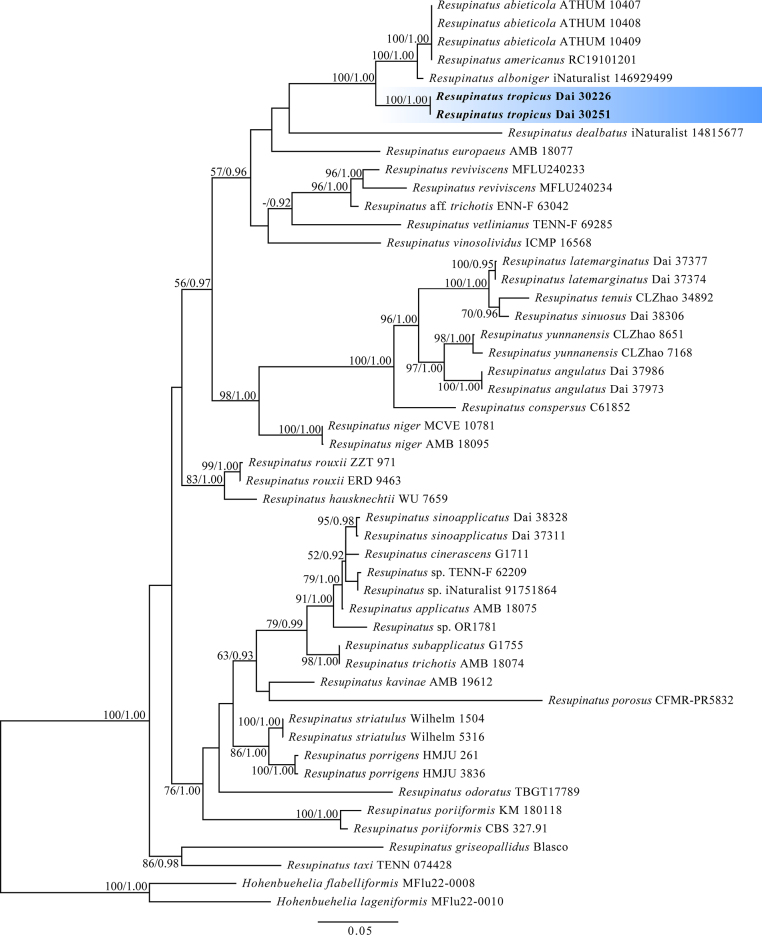
The Maximum Likelihood (ML) tree of *Resupinatus* was conducted based on a combined ITS + nLSU sequences. Maximum Likelihood (ML) bootstrap support values (BS ≥ 50%) and BI posterior probabilities (BPP ≥ 0.9) are indicated on the corresponding branches, while BS ≥ 70% and BPP ≥ 0.95 were considered as significantly supported. The newly described species, *R. tropicus*, is shown in bold. The scale bar (bottom) denotes the number of substitutions per site.

The concatenated ITS + nLSU dataset of *Resupinatus* included sequences from 48 samples representing 32 taxa. A two-locus combined dataset consisted of ITS (1–733 characters) and nLSU (734–1,636 characters), of which 1,070 are constant, 103 are parsimony-uninformative, and 463 are parsimony-informative. The GTRGAMMAIX and GTR + I + G substitution models were selected for the ML and BI analyses, respectively. The BI analysis recovered a tree topology essentially identical to that obtained from the ML analysis, with an average standard deviation of split frequencies of 0.005591, indicating satisfactory convergence. Given the high level of congruence between the two methods, only the BI tree is presented, with branch support values indicated as ML bootstrap support values (BS ≥ 50%) and BI posterior probabilities (BPP ≥ 0.9) in Fig. 1.

Phylogenetic analyses suggested that the two specimens of *Resupinatus tropicus* formed a highly supported lineage (100/1.00). Meanwhile, *R. abieticola*, *R. alboniger*, *R. americanus* Consiglio & Setti, and *R. tropicus* grouped together to form a distinct and fully-supported clade (100% BS, 1.00 BPP).

**Figure 2.**
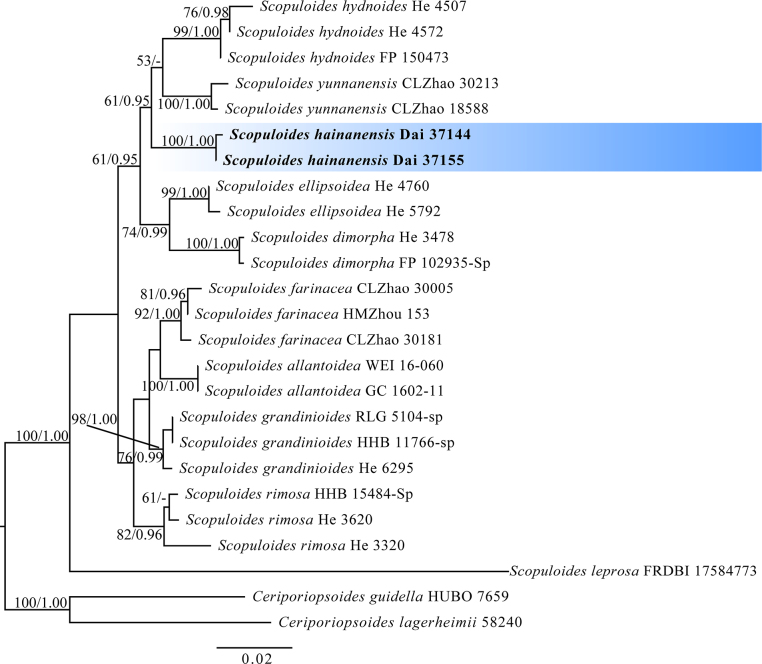
The Maximum Likelihood (ML) tree of *Scopuloides* is conducted based on a combined ITS + nLSU sequences. Maximum Likelihood (ML) bootstrap support values (BS ≥ 50%) and BI posterior probabilities (BPP ≥ 0.9) are indicated on the corresponding branches, while BS ≥ 70% and BPP ≥ 0.95 were considered as significantly supported. The newly described species, *S. hainanensis*, is shown in bold. The scale bar (bottom) denotes the number of substitutions per site.

The concatenated ITS + nLSU dataset of *Scopuloides* included sequences from 23 samples representing ten taxa. A two-locus combined dataset consisted of ITS (1–679 characters) and nLSU (680–2,036 characters), of which 1,764 are constant, 109 are parsimony-uninformative, and 163 are parsimony-informative. The GTRGAMMAIX and GTR + I + G substitution models were selected for the ML and BI analyses, respectively. The BI analysis recovered a tree topology essentially identical to that obtained from the ML analysis, with an average standard deviation of split frequencies of 0.005671, indicating satisfactory convergence. Given the high level of congruence between the two methods, only the BI tree is presented, with branch support values indicated as ML bootstrap support values (BS ≥ 50%) and BI posterior probabilities (BPP ≥ 0.9) in Fig. 2.

Our new species formed a highly supported lineage within *Scopuloides* and is closely related to *S. hydnoides* and *S. yunnanensis* with well-supported values (61/0.95 for BS and BPP), and grouped with *S. ellipsoidea* and *S. dimorpha* with well-supported values (61/0.95 for BS and BPP).

### Taxonomy

#### Resupinatus tropicus

Taxon classificationFungiAgaricalesPleurotaceae

Q.X. Guan, H. Zhao & Y.C. Dai sp. nov.

B95329A6-769D-54F3-A96F-364FB73FB244

MycoBank No: 863828

Figs 3 a, b, 4, 5

##### Holotype.

China • Hainan Province, National Park of Hainan Tropical Rainforest, Baisha County, Qingsong, on fallen angiosperm branch, 19.116649°N, 109.265192°E, elev. 420 m, 24 November 2024, Dai 30226 (BJFC 050485).

**Figure 3.**
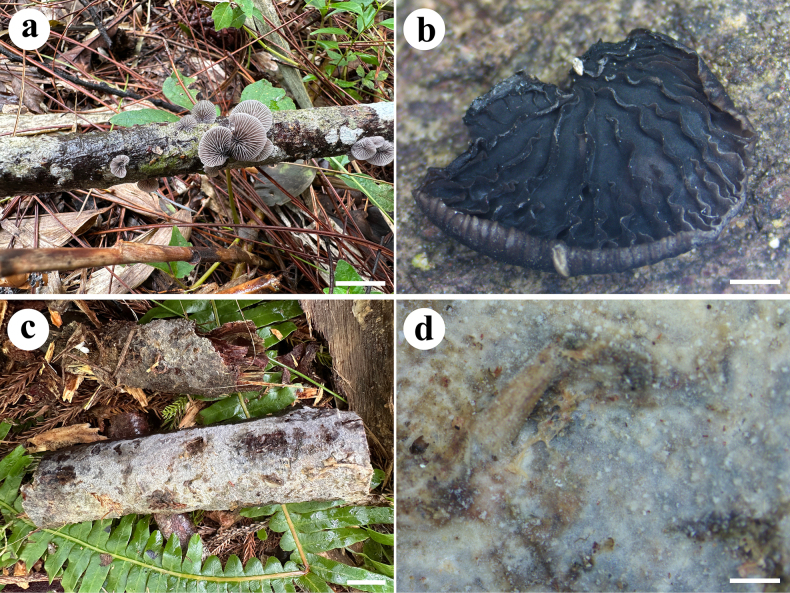
Basidiomata of *Resupinatus tropicus* and *Scopuloides hainanensis*. **a, b**. *R. tropicus* (holotype, Dai 30226); **c, d**. *S. hainanensis* (holotype, Dai 37144). Scale bars: 1 cm (**a, c**); 1 mm (**b, d**).

**Figure 4.**
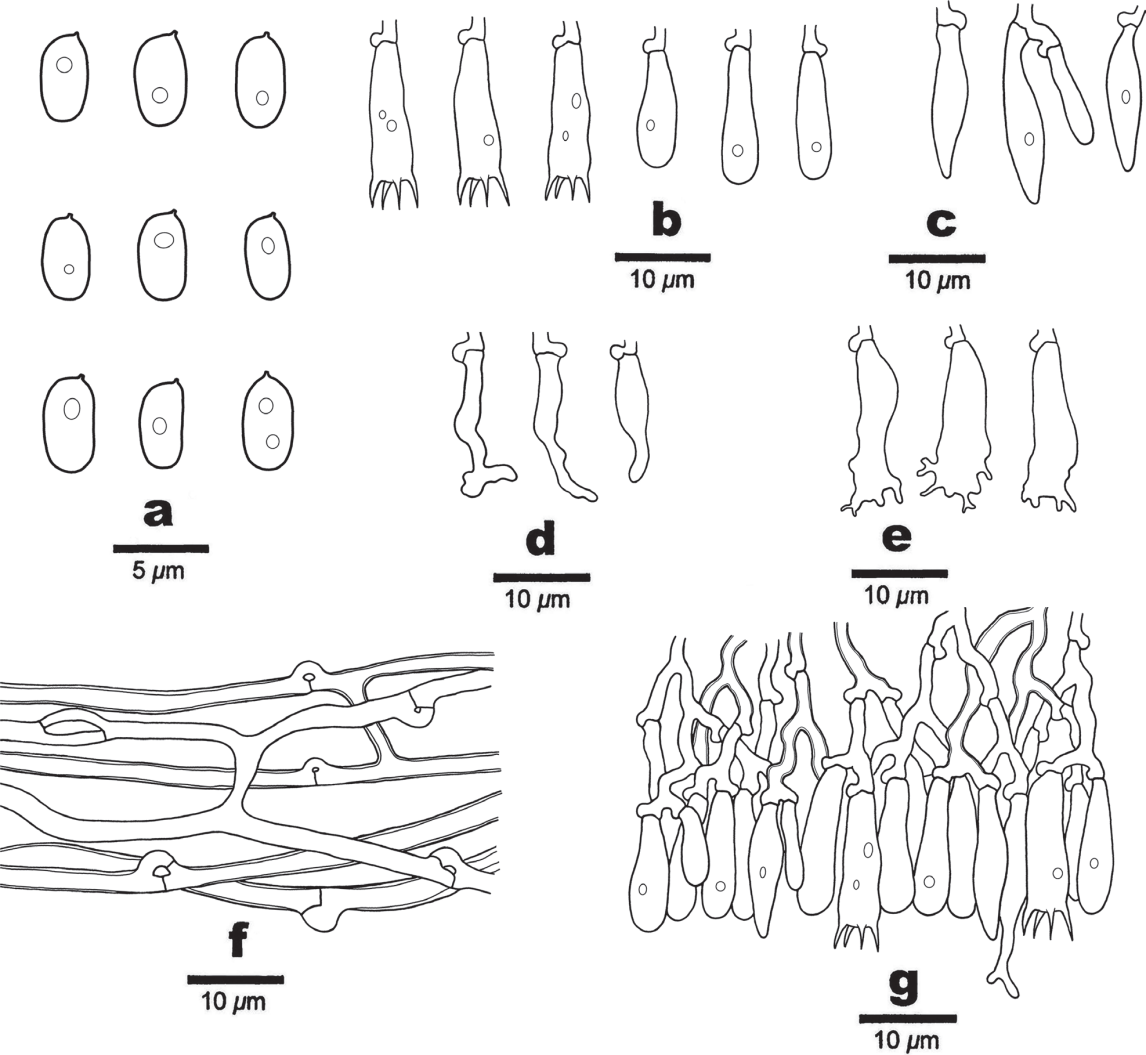
Microscopic structures of *Resupinatus tropicus* (holotype, Dai 30226). **a**. Basidiospores; **b**. Basidia and basidioles; **c, d**. Pleurocystidia; **e**. Cheilocystidia; **f**. Hyphae; **g**. A section of hymenium.

**Figure 5.**
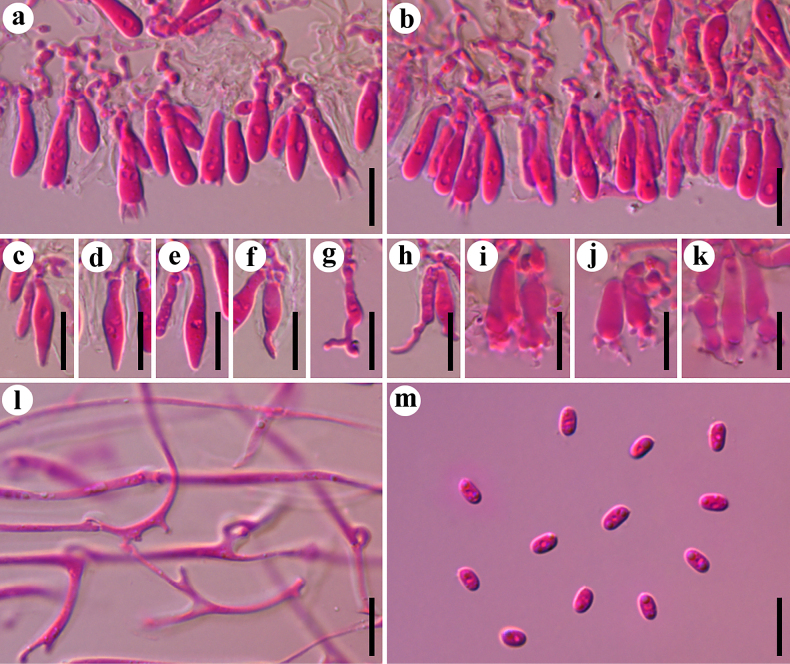
Sections of hymenia of *Resupinatus tropicus* (holotype, Dai 30226). **a, b**. Basidia and basidioles; **c–h**. Pleurocystidia; **i–k**. Cheilocystidia; **l**. Hyphae; **m**. Basidiospores. Scale bars: 10 μm (**a–m**); 10 × 100 Oil.

##### Etymology.

*Tropicus* (Lat.) refers to the new species found from tropical China.

##### Description.

Basidiomata annual, sessile, gelatinous, flabelliform or occasional suborbicular, laterally or dorsally attached to substrate, fragile and watery when fresh, becoming firm-gelatinous when dry. Pileus orbicular when juvenile, becoming flabelliform when mature, upper surface pale mouse gray when fresh, becoming dark gray to black when dry, smooth, up to 10 mm in diam.; margin unrolled to incurved when juvenile, becoming straight, entire. Hymenium lamellate, lamellae with different lengths. Stipe absent. Context dark brown to black, leathery when dry, up to 0.3 mm thick.

Hyphal system monomitic; generative hyphae with clamp connections, colorless, thin- to thick-walled, branched, interwoven, 1–2 µm in diameter. Cheilocystidia clavate to cylindrical with irregular, bifurcate, multifurcate, or rarely nodulose, thin-walled, colorless, 11–16 × 4–6.5 µm, arising from generative hyphae. Pleurocystidia fusiform to with branched apices, 13–18 × 2–4 µm. Basidia clavate, with four sterigmata and a basal clamp connection, with a few guttules, 13–17 × 3.5–4.5 µm; basidioles similar to basidia, but slightly smaller. Basidiospores cylindrical to oblong-ellipsoid, colorless, thin-walled, smooth, with a few guttules, IKI–, CB–, 4.4–5.2(–5.5) × 2.4–2.9 µm, L = 4.78 µm, W = 2.64 µm, Q = 1.81 (n = 30/1).

##### Type of rot.

white rot.

##### Additional specimen examined

**(*paratype*)**. China • Hainan Province, National Park of Hainan Tropical Rainforest, Baisha County, Qingsong, on fallen angiosperm branch, 19.116649°N, 109.265192°E, elev. 420 m, 24 November 2024, Dai 30251 (BJFC 050510).

#### Scopuloides hainanensis

Taxon classificationFungiPolyporalesMeruliaceae

Q.X. Guan, H. Zhao & Y.C. Dai sp. nov.

6711AD46-0C5B-5F3E-B428-9B80F12C5CF1

MycoBank No: 863829

Figs 3 c, d, 6, 7

##### Holotype.

China • Hainan Province, National Park of Hainan Tropical Rainforest, Diaoluo Mountain, on fallen branch of *Dacrydium pectinatum*, 18.725211°N, 109.868396°E, elev. 927 m, 3 July 2025, Dai 37144 (BJFC 058403).

**Figure 6.**
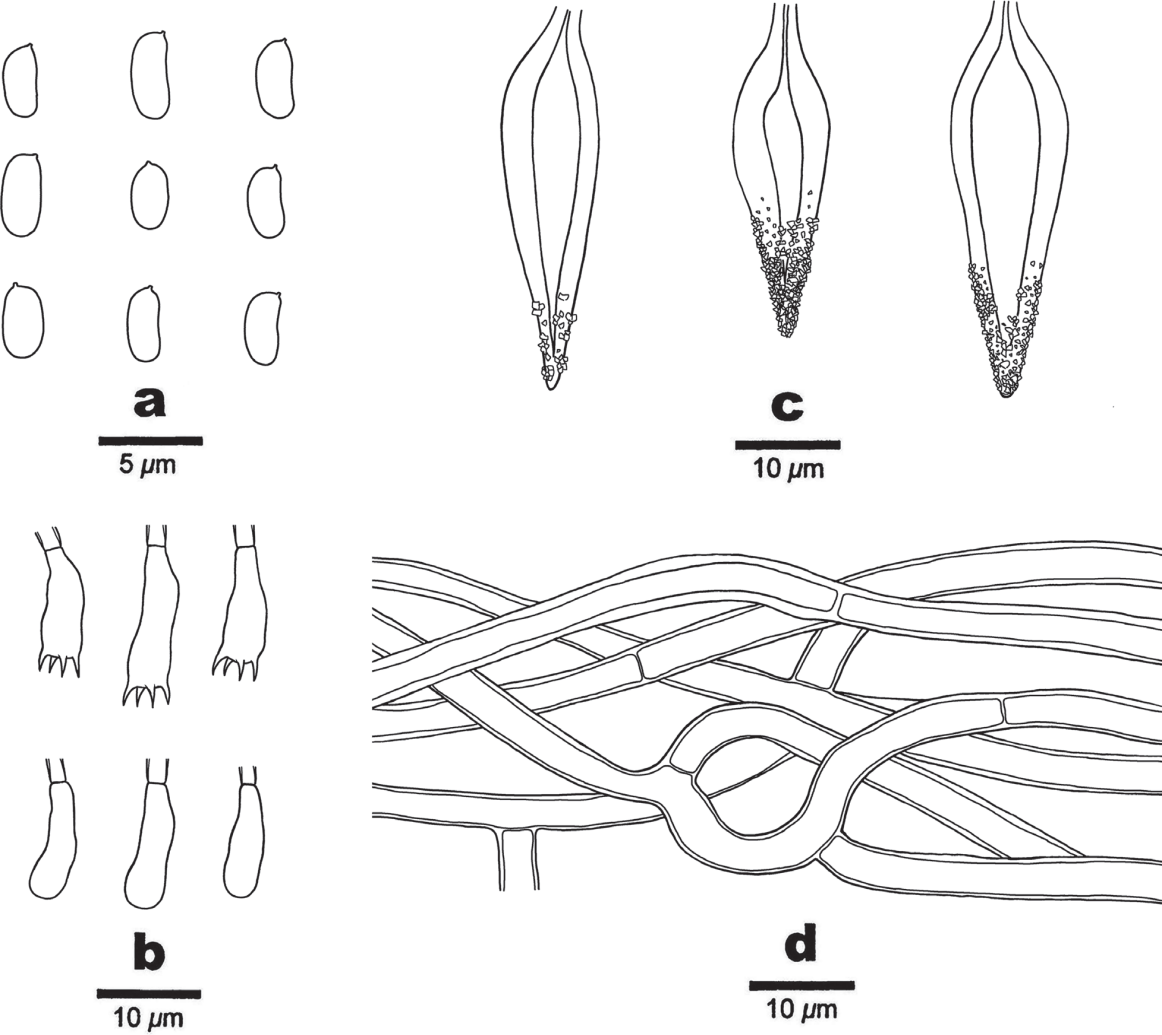
Microscopic structures of *Scopuloides hainanensis* (holotype, Dai 37144). **a**. Basidiospores; **b**. Basidia and basidioles; **c**. Lamprocystidia; **d**. Hyphae.

**Figure 7.**
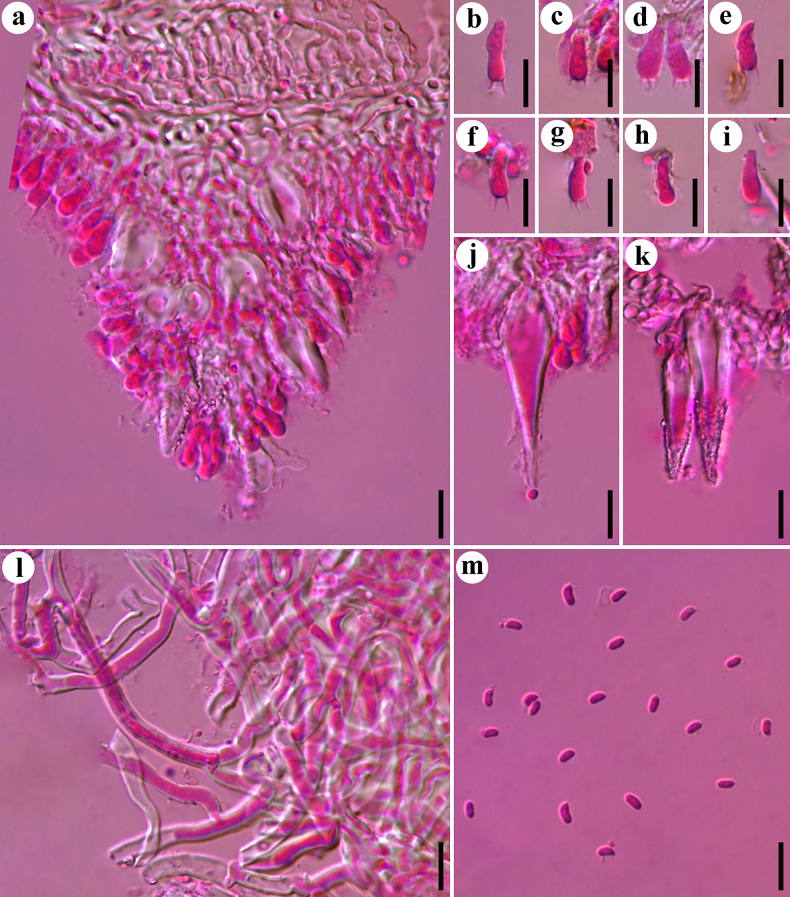
Sections of hymenium of *Scopuloides hainanensis* (holotype, Dai 37144). **a**. A section of hymenium; **b–i**. Basidia and basidioles; **j, k**. Lamprocystidia; **l**. Hyphae; **m**. Basidiospores. Scale bars: 10 μm (**a–m**); 10 × 100 Oil.

##### Etymology.

*Hainanensis* (Lat.) refers to the species collected from Hainan Province, China.

##### Description.

Basidiomata annual, resupinate, closely adnate, membranaceous, without odor or taste when fresh, difficult to separate from substrate, up to 15 cm long, 5 cm wide, 0.3 mm thick. Hymenial surface grandinioid, white to gray when fresh, turning to gray upon drying. Margin gradually thinning out, concolorous with the hymenial.

Hyphal system monomitic; generative hyphae with simple septa, colorless, thick-walled, branched, interwoven, 2.5–3.5 μm in diameter, IKI–, CB–; tissues unchanged in KOH. Lamprocystidia abundant, subulate to fusiform, colorless, thick-walled, 25–45 × 7.5–11 µm. Basidia clavate, with four sterigmata and a basal simple septum, 9–12 × 3–4 µm; basidioles numerous, in shape similar to basidia but smaller. Basidiospores subcylindrical to allantoid, colorless, thin-walled, smooth, IKI–, CB–, 3.4–4.4 × 1.6–2.1 µm, L = 3.89 µm, W = 1.81 µm, Q = 2.06–2.23 (n = 60/2).

##### Type of rot.

white rot.

##### Additional specimen examined

**(*paratype*)**. China • Hainan Province, National Park of Hainan Tropical Rainforest, Diaoluo Mountain, on fallen angiosperm branch, 18.725211°N, 109.868396°E, elev. 927 m, 3 July 2025, Dai 37155 (BJFC 058414).

## Discussion

China, spanning a wide latitudinal and environmental gradient from boreal forests to tropical rainforests, provides an ideal natural framework for examining large-scale patterns of fungal diversity, especially in subtropical to tropical regions (Zhao et al. 2024; Bao et al. 2025; Zeng et al. 2026; Zhang et al. 2026). Hainan Province, as a tropical region in China, is where many new species have been discovered in recent years (Dai et al. 2021; Zhao et al. 2025a). Large-scale macrofungi diversity surveys also indicate that Hainan harbors riches wood-decaying fungal species diversity, particularly within Agaricales, Hymenochaetales, and Polyporales (Ma et al. 2022). During our ongoing investigation of macrofungi, we have identified two new species of wood-decaying fungi belonging to Agaricales and Polyporales in Hainan, suggesting that the region still has a high potential for fungal species diversity and requires further in-depth, comprehensive, and sustained surveys.

In the present study, we describe two new species, *Resupinatus tropicus* and *Scopuloides hainanensis*, based on phylogenetic analyses and morphological characteristics. Phylogenetically, the new species *R. tropicus* formed a distinct, robustly supported lineage, and grouped with *R. abieticola*, *R. alboniger*, and *R. americanus* in a highly supported clade (100/1.00). However, *R. abieticola* and *R. alboniger* are distinguished from *R. tropicus* by larger basidia (15–23 × 4–7 μm in *R. abieticola*, 17–22 × 5–6 μm in *R. alboniger* vs. 13–17 × 3.5–4.5 µm), bigger basidiospores (5–7.8 × 2.4–3.6 μm in *R. abieticola*, 5.5–6.6 × 3–4 μm in *R. alboniger* vs. 4.4–5.2 × 2.4–2.9 µm), and larger cheilocystidia (13–27.5 × 5.5–9 μm in *R. abieticola*, 20–28 × 5–8 μm in *R. alboniger* vs. 11–16 × 4–6.5 µm; Singer 1978; Triantafyllou et al. 2026). In addition, specimens of *R. abieticola* and *R. americanus* formed a highly supported independent lineage in our phylogeny (100/1.00, Fig. 1), which is consistent with the suggestion of Triantafyllou et al. (2026) that these specimens belong to *R. abieticola*.

Morphologically, *Resupinatus* species can be divided into two groups, one group having sessile basidiomata with a lamellate hymenophore and the other having resupinate basidiomata with a poroid or cyphelloid hymenophore. The new species *R. tropicus* may be confused with *R. alboniger*, *R. applicatus*, *R. porrigens*, *R. sinoapplicatus*, and *R. trichotis* by sessile basidiomata with lamellate hymenophore and it is also distributed in China. However, *R. applicatus*, *R. porrigens*, *R. sinoapplicatus*, and *R. trichotis* are distinguished from *R. tropicus* by having globose to subglobose basidiospores (Li and Bau 2014; Liu et al. 2024; Zhuang et al. 2026b); *R. alboniger* differs from *R. tropicus* by larger cheilocystidia (20–28 × 5–8 µm vs. 11–16 × 4–6.5 µm), larger basidia (17–22 × 5–6 µm vs. 13–17 × 3.5–4.5 µm), and larger basidiospores (5.5–6.6 × 3–4 µm vs. 4.4–5.2 × 2.4–2.9 µm; Li and Bau 2014).

Phylogenetically, the new species *S. hainanensis* formed a highly supported lineage within *Scopuloides* and is closely related to *S. hydnoides* and *S. yunnanensis* with well-supported values (61/0.95, Fig. 2), but the latter two species differ from *S. hainanensis* by the presence of septate cystidia (Hjortstam and Ryvarden 1979; Gu et al. 2024).

Morphologically, the new species *Scopuloides hainanensis* is similar to *S. ellipsoidea* and *S. farinacea* by sharing a grandinioid hymenial surface and having only one type of lamprocystidia, but the latter two species differ from *S. hainanensis* in having smaller basidiospores (2.6–3.1 × 1.5–1.8 µm in *S. ellipsoidea*, 2.8–3.5 × 1.4–2 µm in *S. farinacea* vs. 3.4–4.4 × 1.6–2.1 µm; Gu et al. 2025).

### Key to known species of *Resupinatus* in China

**Table:** 

1	Basidiomata sessile, with lamellate hymenophore	**2**
–	Basidiomata resupinate, with poroid or cyphelloid hymenophore	**7**
2	Pileus surface pale mouse gray	**3**
–	Pileus surface red-brown, grayish brown to black-brown or black	**4**
3	Basidiospores globose, 3.9–5 × 3.7–4.7 µm	***R. sinoapplicatus***
–	Basidiospores reniform to oblong-ellipsoid, 4.4–5.2 × 2.4–2.9 µm	***R. tropicus***
4	Pileus surface red-brown toward the margin	***R. porrigens***
–	Pileus surface grayish brown to black-brown or black	**5**
5	Basidiospores oblong ellipsoid, 5.5–6.6 × 3–4 µm	***R. alboniger***
–	Basidiospores globose to subglobose	**6**
6	Pileus surface yellow to brown tomentose; cystidioles > 20 µm in length	***R. applicatus***
–	Pileus surface black tomentose; cystidioles < 20 µm in length	***R. trichotis***
7	Basidiomata with angular or sinuous to irregular pores	**8**
–	Basidiomata with circular to subcircular pores	**9**
8	Cystidioles 17–22 × 3.5–6.5 µm; basidiospores ellipsoid, 5.3–6.6 × 4–5.2 µm	***R. sinuosus***
–	Cystidioles 10–15 × 3–5.5 µm; basidiospores cylindrical to allantoid, 5.6–7.3 × 2.6–3.6 µm	***R. angulatus***
9	Pores > 7 per mm; basidia > 8.5 µm in width	***R. tenuis***
–	Pores < 7 per mm; basidia < 8.5 µm in width	**10**
10	Cystidioles absent; crystal encrusted branched hyphae with short finger-like present	***R. yunnanensis***
–	Cystidioles present; crystal encrusted branched hyphae absent	***R. latemarginatus***

### Key to known species of *Scopuloides* in China

**Table:** 

1	Hymenial surface grandinioid	**2**
–	Hymenial surface odontioid	**6**
2	With one type of cystidia	**3**
–	With two types of cystidia	**5**
3	Basidiospores usually > 3.5 µm in length	***S. hainanensis***
–	Basidiospores usually < 3.5 µm in length	**4**
4	Basidia 10–18 × 3–4 µm	***S. ellipsoidea***
–	Basidia 4.6–8 × 1.8–2.7 µm	***S. farinacea***
5	Lamprocystidia > 40 µm in length; septate cystidia > 50 µm in length	***S. grandinioides***
–	Lamprocystidia < 40 µm in length; septate cystidia < 50 µm in length	***S. yunnanensis***
6	With one type of cystidia	***S. rimosa***
–	With two types of cystidia	**7**
7	Basidiospores short-allantoid, > 1.8 µm in width	***S. hydnoides***
–	Basidiospores ellipsoid, < 1.8 µm in width	**8**
8	Basidiospores 3.3–3.7 × 1.2–1.4 µm	***S. allantoidea***
–	Basidiospores 3–3.2 × 1.2–1.8 µm	***S. dimorpha***

## Supplementary Material

XML Treatment for Resupinatus tropicus

XML Treatment for Scopuloides hainanensis

## Additional information

### Artificial Intelligence (AI) use

The authors accept full responsibility for the content of the manuscript, including the disclosure of any use of AI.

No AI tools were used in the preparation of this manuscript.

### Author ORCIDs

Q.-X. Guan https://orcid.org/0000-0002-7072-080X

H. Zhao https://orcid.org/0000-0003-2938-5613

F. Wu https://orcid.org/0000-0002-1455-6486

X. Zhang https://orcid.org/0009-0005-8363-7852

Y.-C. Dai https://orcid.org/0000-0002-6523-0320

A.-H. Zhu https://orcid.org/0000-0002-2812-8108

### Data availability

All of the data that support the findings of this study are available in the main text or Supplementary Information.

## Supplementary materials

Supplementary material 1 A list of species, specimens, and GenBank accession numbers of sequences used in this study Data type docx Explanation note “–” refers to the unavailability of the data. https://binary.pensoft.net/file/1665316This dataset is made available under the Open Database License (http://opendatacommons.org/licenses/odbl/1.0/). The Open Database License (ODbL) is a license agreement intended to allow users to freely share, modify, and use this Dataset while maintaining this same freedom for others, provided that the original source and author(s) are credited. Qian-Xin Guan, Heng Zhao, Fang Wu, Xin Zhang, Yu-Cheng Dai, An-Hong Zhu
