# Supplementary material for: Two new wood-decaying fungi, Resupinatus tropicus and Scopuloides hainanensis (Agaricomycetes, Basidiomycota) from Hainan Province, southern China
Source: MycoKeys. 2026 Jun 1;133:149–67. doi: 10.3897/mycokeys.133.198088 (PMC13247632; doi:10.3897/mycokeys.133.198088)
Supplement: Supplementary material 1 — A list of species, specimens, and GenBank accession numbers of sequences used in this study [file mycokeys-133-149-s001.docx]

Table S1. The GenBank accession numbers of sequences used in this study. – refers to the unavailability of the data. New species are shown in bold.

| Species name | Voucher No. | GenBank Accession no. | |
| --- | --- | --- | --- |
|  |  | ITS | nLSU |
| *Ceriporiopsoides guidella* | HUBO 7659 | FJ496687 | FJ496722 |
| *Ceriporiopsoides lagerheimii* | 58240 | KX008365 | KX081077 |
| *Hohenbuehelia flabelliformis* | MFlu22 0008 | OP236779 | OM521957 |
| *Hohenbuehelia lageniformis* | MFlu22 0010 | OP236781 | OM521958 |
| *Resupinatus abieticola* | ATHUM 10407 | PX057674 | PX057680 |
| *Resupinatus abieticola* | ATHUM 10408 | PX057675 | PX057681 |
| *Resupinatus abieticola* | ATHUM 10409 | PX057676 | PX057682 |
| *Resupinatus* aff*. trichotis* | ENN F 63042 | KP026231 | – |
| *Resupinatus alboniger* | iNaturalist 146929499 | OR081334 | – |
| *Resupinatus americanus* | RC19101201 | OM397444 | – |
| *Resupinatus angulatus* | Dai 37973 | PX982928 | PX982942 |
| *Resupinatus angulatus* | Dai 37986 | PX982929 | PX982943 |
| *Resupinatus applicatus* | AMB 18075 | KU355368 | KU355411 |
| *Resupinatus cinerascens* | G1711 | – | MK278551 |
| *Resupinatus conspersus* | C61852 | AY571061 | AY571024 |
| *Resupinatus dealbatus* | iNaturalist 14815677 | ON416908 | – |
| *Resupinatus europaeus* | AMB 18077 | KU355366 | KU355410 |
| *Resupinatus griseopallidus* | Blasco | MG553642 | MG553649 |
| *Resupinatus hausknechtii* | WU 7659 | KU355370 | KU355412 |
| *Resupinatus kavinae* | AMB 19612 | MG553643 | MG553650 |
| *Resupinatus latemarginatus* | Dai 37374 | PX982934 | PX982948 |
| *Resupinatus latemarginatus* | Dai 37377 | PX982935 | PX982949 |
| *Resupinatus niger* | AMB 18095 | KU355371 | KU355413 |
| *Resupinatus niger* | MCVE 10781 | KU355372 | KU355414 |
| *Resupinatus odoratus* | TBGT17789 | MT452498 | – |
| *Resupinatus poriiformis* | CBS 327.91 | AY571062 | AY571025 |
| *Resupinatus poriiformis* | KM 180118 | MZ159469 | – |
| *Resupinatus porosus* | CFMR PR5832 | NR_119556 | – |
| *Resupinatus porrigens* | HMJU 261 | NR_198332 | NG_243157 |
| *Resupinatus* *porrigens* | HMJU 3826 | OP729420 | OP729419 |
| *Resupinatus reviviscens* | MFLU240233 | PQ036937 | PQ036942 |
| *Resupinatus reviviscens* | MFLU240234 | PQ036938 | PQ036943 |
| *Resupinatus rouxii* | ERD 9463 | OP289290 | – |
| *Resupinatus rouxii* | ZZT 971 | MH168326 | MH190787 |
| *Resupinatus sinoapplicatus* | Dai 37311 | PX982936 | PX982950 |
| *Resupinatus sinoapplicatus* | Dai 37328 | PX982937 | – |
| *Resupinatus sinuosus* | Dai 38306 | PX982938 | PX982951 |
| *Resupinatus* sp. | iNaturalist 91751864 | OQ389423 | – |
| *Resupinatus* sp. | OR1781 | PQ475810 | PQ036943 |
| *Resupinatus* sp. | TENN-F 62209 | KP026229 | – |
| *Resupinatus striatulus* | Wilhelm 1504 | KU355374 | – |
| *Resupinatus striatulus* | Wilhelm 5316 | MH137831 | MH169342 |
| *Resupinatus subapplicatus* | G1755 | – | MK278557 |
| *Resupinatus taxi* | TENN 074428 | MH558280 | – |
| *Resupinatus tenuis* | CLZhao 34892 | PV197932 | PV197946 |
| *Resupinatus trichotis* | AMB 18074 | KU355378 | KU355416 |
| ***Resupinatus tropicus*** | **Dai 30226** | **PZ381633** | **PZ381637** |
| ***Resupinatus tropicus*** | **Dai 30251** | **PZ381634** | **PZ381638** |
| *Resupinatus vetlinianus* | TENN F69285 | KP026243 | KP987309 |
| *Resupinatus vinosolividus* | ICMP 16568 | MZ325958 | – |
| *Resupinatus yunnanensis* | CLZhao 7168 | OP901838 | – |
| *Resupinatus yunnanensis* | CLZhao 8651 | OP901839 | OP904197 |
| *Scopuloides allantoidea* | WEI 16-060 | MZ637081 | MZ637279 |
| *Scopuloides allantoidea* | GC 1602-11 | MZ637080 | MZ637278 |
| *Scopuloides dimorpha* | He 3478 | PP549566 | PP549600 |
| *Scopuloides dimorpha* | FP 102935-Sp | KP135353 | KP135285 |
| *Scopuloides ellipsoidea* | He 4760 | PP549569 | PP549603 |
| *Scopuloides ellipsoidea* | He 5792 | PP549570 | PP549604 |
| *Scopuloides farinacea* | CLZhao 30005 | PV470558 | PV474203 |
| *Scopuloides farinacea* | HMZhou 153 | PV470560 | PV474205 |
| *Scopuloides farinacea* | CLZhao 30181 | PV470559 | PV474204 |
| *Scopuloides grandinioides* | RLG 5104-sp | KP135351 | KP135283 |
| *Scopuloides grandinioides* | HHB 11766-sp | KP135348 | – |
| *Scopuloides grandinioides* | He 6295 | PP549571 | PP549605 |
| ***Scopuloides hainanensis*** | **Dai 37144** | **PZ381635** | **PZ381639** |
| ***Scopuloides hainanensis*** | **Dai 37155** | **PZ381636** | **PZ381640** |
| *Scopuloides hydnoides* | He 4507 | PP549572 | PP549606 |
| *Scopuloides hydnoides* | He 4572 | PP549573 | PP549607 |
| *Scopuloides hydnoides* | FP 150473 | KP135355 | KP135284 |
| *Scopuloides leprosa* | FRDBI 17584773 | MW487975 | – |
| *Scopuloides rimosa* | HHB 15484-Sp | KP135352 | KP135281 |
| *Scopuloides rimosa* | He 3620 | PP549575 | PP549609 |
| *Scopuloides rimosa* | He 3320 | PP549574 | PP549608 |
| *Scopuloides yunnanensis* | CLZhao 30213 | PP511314 | PP511317 |
| *Scopuloides yunnanensis* | CLZhao 18588 | PP511312 | PP511315 |
